# Personalized, interdisciplinary patient pathway for cross-sector care of multimorbid patients (eliPfad trial): study protocol for a randomized controlled trial

**DOI:** 10.1186/s13063-024-08026-8

**Published:** 2024-03-11

**Authors:** Christoph Heinrich Lindemann, Volker Burst, Linus Alexander Völker, Sebastian Brähler, Dusan Simic, Ingrid Becker, Martin Hellmich, Clarissa Kurscheid, Nadine Scholten, Ruben Krauspe, Kerstin Leibel, Stephanie Stock, Paul Thomas Brinkkoetter

**Affiliations:** 1 Department II of Internal Medicine and Center for Molecular Medicine Cologne (CMMC), Faculty of Medicine, University of Cologne, University Hospital Cologne, Cologne, Germany; 2 Cologne Institute for Health Economics and Clinical Epidemiology, University of Cologne, Faculty of Medicine and University Hospital Cologne, Cologne, Germany; 3 Institute of Medical Statistics and Computational Biology, Faculty of Medicine and University Hospital Cologne, University of Cologne, Cologne, Germany; 4 Research Institute for Health and System Development, EUFH University of Applied Sciences, Cologne, Germany; 5 Faculty of Medicine and University Hospital Cologne, Institute of Medical Sociology, Health Services Research, and Rehabilitation Science, Chair for Health Services Research, University of Cologne, Cologne, Germany

**Keywords:** Geriatrics, e-health, Multimorbidity, Chronic diseases, Case management, Rehospitalization, Health services research, Study protocol, Randomized controlled trial

## Abstract

**Background:**

Multimorbid and frail elderly patients often carry a high burden of treatment. Hospitalization due to the onset of an acute illness can disrupt the fragile balance, resulting in further readmissions after hospital discharge. Current models of care in Germany do not meet the needs of this patient group. Rather lack of coordination and integration of care combined with a lack of interdisciplinary approaches result in fragmented and inadequate care and increase the burden of treatment even more.

**Methods:**

eliPfad is a randomized controlled trial conducted in 6 hospitals in Germany. Multimorbid elderly patients aged 55 or older are randomly assigned to the intervention or control group. Patients in the intervention group receive the eliPfad intervention additional to standard care. The core components of eliPfad are:

- Early assessment of patients’ individual treatment burden and support through a specially trained case manager
- Involvement of the patient’s general practitioner (GP) right from the beginning of the hospital stay
- Preparation of an individual, cross-sectoral treatment plan through the interdisciplinary hospital team with the involvement of the patient’s GP
- Establishment of a cross-sectoral electronic patient record (e-ePA) for documentation and cross-sectoral exchange
- Support/Promote patient adherence
- Tailored early rehabilitation during the hospital stay, which is continued at home
- Close-tele-monitoring of medically meaningful vital parameters through the use of tablets, digital devices, and personal contacts in the home environment

The intervention period begins in the hospital and continues 6 weeks after discharge. Patients in the control group will be treated according to standard clinical care and discharged according to current discharge management. The primary aim is the prevention/reduction of readmissions in the first 6 months after discharge. In addition, the impact on health-related quality of life, the burden of treatment, survival, self-management, medication prescription, health literacy, patient-centered care, cost-effectiveness, and process evaluation will be examined. Nine hundred forty-eight patients will be randomized 1:1 to intervention and control group.

**Discussion:**

If eliPfad leads to fewer readmissions, proves (cost-)effective, and lowers the treatment burden, it should be introduced as a new standard of care in the German healthcare system.

**Trial registration:**

The trial was registered in the German Clinical Trials Registry (Deutsches Register Klinischer Studien (DRKS)) on 08/14/2023 under the ID DRKS00031500.

## Administrative information

Note: The numbers in curly brackets in this protocol refer to SPIRIT checklist item numbers. The order of the items has been modified to group similar items (see http://www.equator-network.org/reporting-guidelines/spirit-2013-statement-defining-standard-protocol-items-for-clinical-trials/).

**Table:** 

Title {1}	Personalized, interdisciplinary patient pathway for cross-sectoral care of multimorbid patients (eliPfad trial): study protocol for a randomized controlled trial
Trial registration {2a and 2b}.	The trial was registered in the German Clinical Trials Registry (Deutsches Register Klinischer Studien (DRKS)) on 08/14/2023 under the ID DRKS00031500.
Protocol version {3}	Date: 08/10/2023. Version: Studienprotokoll, Version 1.1
Funding {4}	The entire trial and its conceptualization are funded by the innovation fund of the Federal Joint Committee (Gemeinsamer Bundesausschuss (G-BA)). https://innovationsfonds.g-ba.de/projekte/neue-versorgungsformen/elipfad-personalisierter-interdisziplinaerer-patientenpfad-zur-sektorenuebergreifenden-versorgung-multimorbider-patienten-mit-telemedizinischem-monitoring.507
Author details {5a}	Lindemann, Christoph Heinrich (1,6); Burst, Volker (1,6); Völker, Linus Alexander (1); Brähler, Sebastian (1); Simic, Dusan (2); Becker, Ingrid (3); Hellmich, Martin (3); Kurscheid, Clarissa (4); Scholten, Nadine (5); Krauspe, Ruben (2); Leibel, Kerstin (2); Stock, Stephanie (2,6); Brinkkoetter, Paul Thomas (1,6) 1) Department II of Internal Medicine and Center for Molecular Medicine Cologne (CMMC), University of Cologne, Faculty of Medicine and University Hospital Cologne, Cologne, Germany 2) Cologne Institute for Health Economics and Clinical Epidemiology, University of Cologne, Faculty of Medicine and University Hospital Cologne, Cologne, Germany 3) Institute of Medical Statistics and Computational Biology, Faculty of Medicine and University Hospital Cologne, University of Cologne, Cologne, Germany 4) Research Institute for Health and System Development, EUFH University of Applied Sciences, Cologne, Germany 5) Faculty of Medicine and University Hospital Cologne, Institute of Medical Sociology, Health Services Research, and Rehabilitation Science, Chair for Health Services Research, University of Cologne, Cologne, Germany 6) These authors contributed equally
Name and contact information for the trial sponsor {5b}	The trial sponsor is the University of Cologne, represented by the Department II of Internal Medicine of the University Hospital Cologne. The postal address is: Department II for Internal Medicine, Kerpener Straße 62, 50937 Köln The trial funder is the G-BA, the highest decision-making public health agency in Germany. The postal address is: Gemeinsamer Bundesausschuss (G-BA), Gutenbergstraße 13, 10587 Berlin
Role of sponsor {5c}	The trial sponsor is primarily responsible for the study design as well as the collection and management of the data. The writing of the report or the decision to submit the report for publication is also the responsibility of the sponsor. However, analysis and interpretation of the data is carried out by independent evaluators. The trial funder is not involved in and has no ultimate authority over the study design as well as the collection, management, analysis, and interpretation of the data. The same applies to the writing of the report or the decision to submit the report for publication.

## Introduction

### Background and rationale {6a}

#### Background

The category of the elderly is one of the fastest growing patient groups in Germany. A substantial number of these patients is characterized by multimorbidity and frailty [1]. Frailty is described as a functional decrease across multiple physiological factors, such as loss of skeletal muscle mass and strength [2]. As a result, frail patients display a decreased ability to cope with stressors such as a change in environment through transition to hospital. Such a stressor may be responsible for patients to decompensate, i.e., lose their everyday functionality and independence [3–5], ultimately giving rise to the need for long-term care. Care of these patients is predominantly characterized by polypharmacy [6], fragmented care, and repeated hospital stays [7]. Predictors for these unplanned readmissions are sociodemographic factors like higher age, multimorbidity, and certain diagnoses such as heart failure, coronary heart disease, chronic kidney disease, and chronic obstructive pulmonary disease (COPD) [8–10]. The main treatment goal for these patients should be to preserve their autonomy and quality of life by avoiding rehospitalization, as recurring hospital stays have been shown to cause undesirable medical consequences such as delirium or falls [11] and economic effects in terms of higher health insurance expenses [3].

Generally, care for elderly patients in Germany is provided through general practitioners (GP) in the ambulatory sector and departments specialized in geriatrics in the inpatient sector. Additionally, numerous differently specialized physicians, inpatient and outpatient, are often involved in their treatment. Both health care systems and self-management of chronic disease demand a high personal investment from the frail elderly. Personal investment, however, requires a certain degree of capacity and capability, which many of the affected frail patients lack [12]. The so-called “burden of treatment,” which consists of the treatment burden (e.g., polypharmacy, appointments), and the burden associated with the condition (symptoms, physical limitations), often exceeds the capacity the patient is able to muster [13]. This overburdening together with other factors such as age, poor functional status prior to admission, or malnutrition often results in repeated, unplanned, and prolonged hospital stays [14, 15]. Thus, measures to decrease the burden of treatment are needed.

The concept of minimally disruptive medicine (MDM) belongs to the wider framework of patient centered care. It aims to ameliorate current problems in the treatment of patients with chronic diseases, such as poor adherence due to a high burden of therapy and lack of coordination and integration of care across sectors, by implementing individually designed treatment plans [7]. This approach considers the patient’s mental and physical capacity and adjusts the personal treatment plan to be coherent with the patient’s capabilities.

A well-known evidence-based framework for patient-centered care for chronically ill is the Chronic Care Model (CCM) [16]. Its overarching goal is to facilitate a productive interaction between activated patients and a proactive care team along with an interdisciplinary and intersectoral cooperation between all health care providers. An activated patient has the confidence, knowledge, and skills to self-manage their own condition. As a result of patient activation, the patient’s self-management improves and ultimately leads to better clinical outcomes as shown for various chronic diseases [17, 18]. The purpose of proactive care is to enable caregivers to identify imminent exacerbations early on and take necessary steps before irreversible consequences occur. Proactive care in the context of frailty can be understood as risk management, where risk is evaluated through a comprehensive geriatric assessment. An interdisciplinary team can then provide the individual and personalized support needed [19]. Previous studies have examined the effects of proactive, integrated, and patient-centered care [19–22] on clinical outcomes among frail elderly. While Berntsen et al. [19] and Uittenbroek et al. [22] report lower emergency care utilization in Norway and better quality of care in The Netherlands, respectively, other studies yielded no significant effect, leaving space for optimized and more comprehensive interventions.

Currently, care structures that address the abovementioned aspects are lacking in Germany. This leads to a high percentage of unplanned hospital readmissions shortly after the initial discharge of up to 57.2% (95% CI 51.4–63.1%) [23]. Similar results have been reported in Australia (38.7%) and Denmark (48.0%) [24, 25]. eliPfad attempts to address this issue by implementing a complex intervention.

#### Rationale

The focus of eliPfad is on implementing patient-centered, proactive, interdisciplinary, cross-sectoral, and minimally disruptive care for frail elderly people with chronic multimorbidity to prevent repeated unplanned readmissions. As the theoretical framework serves an adjusted version of the CCM, which includes aspects of the concept of MDM [7, 16, 26, 27]. To support self-management and activate patients for their treatment, they will receive a “smart assistant” in the form of a tablet, which contains patient education material on coping with the disease to improve health literacy [28]. It furthermore contains medication reminders to improve medication adherence as well as videos with tailored physiotherapeutic exercises as an early rehabilitation measure. Previous research has shown benefiting effects of additional and individual physiotherapy on physical functionality for both hospitalized and community-dwelling frail elderly [29–31]. Besides supporting self-management and intensified physiotherapy, the smart assistant requires patients to daily measure variables such as blood pressure, weight, or pain. This allows for early detection of deterioration and supports proactive care. Telemedical monitoring has been shown to effectively increase both the patients’ and health care professionals’ satisfaction with care [32] as well as to improve clinical outcomes among frail and chronically ill elderly [33, 34]. Patient-centered care is implemented through individualized treatment plans with personal therapy goals. The personal therapy goals are elicited in accordance with the tenets of shared decision-making [35], based on the needs, wishes, and capacity of the patient. The MDM concept is the background for the development of the individual treatment plan. Besides addressing the medical needs of the acute condition, the treatment plan includes aspects of early rehabilitation such as individual physiotherapy and other supportive services to maintain or restore patients’ autonomy and to counteract hospital-induced loss of physical functioning [7]. An individualized treatment plan and a mutually agreed personal treatment goal as part of a patient-centered care intervention has been indicated to improve health care and clinical outcomes [19, 36].

Proactive care is planned based on evidence-based guidelines and enacted both via individualized care plans, monitoring of vital parameters with individual target ranges, and “eliBoards.” The implementation of online “eliBoards” conferences and an electronic patient file allow for close interaction between hospital and GPs, assuring interdisciplinary and cross-sectoral cooperation. Ideally, the GP is involved in the planning of the treatment plan from an early stage on while the patient is still in hospital. This efficient exchange of information aims to ensure that emerging health problems of participating patients are realized and addressed quickly, as shown crucial for successful health care [37]. This form of collaboration between health care sectors using digital means has, to the best of our knowledge, not been done before. After discharge, the developed treatment plan is seamlessly transferred to the ambulatory sector. Patients continue their physiotherapeutic exercises at home and measure relevant vital parameters. The data are captured electronically and transmitted to the patient file in which both a specially trained case manager and GP monitor them on a regular basis. The case manager is furthermore involved in the development of the individual treatment plan and serves as a personal contact for the patients, making the case management a crucial and supporting intersection of patient-centered and proactive care, as shown effective before [19, 22].

eliPfad is expected to lead to an improvement or recovery of the patient’s autonomy and quality of life. Furthermore, an overall reduction of unplanned readmissions to hospitals and rehabilitation measures is expected. The effect of the single components used in eliPfad, i.e., proactive, integrated, and patient-centered care, telemedical support for both patients and caregivers, and extensive physiotherapy across inpatient and outpatient settings, has been proven before. eliPfad uniquely uses these evidence-based intervention components and embeds them in a cross-sectoral complex intervention for frail multimorbid elderly and might therefore improve care of the target population with expected stable costs at the same time.

### Objectives {7}

#### Primary hypothesis

eliPfad reduces the number of unplanned readmissions of multimorbid, elderly patients after the index hospital stay.

#### Secondary hypotheses

The outcome evaluation will be complemented by secondary hypotheses assigned to four different levels: (1) the patient level; (2) the health care system level; (3) the health economics level; and (4) the process evaluation level.

On the patient level, the impact of eliPfad on the following variables will be investigated:

1. Clinical outcomes (vital parameters and clinical chemistry)
2. Depression (Geriatric Depression Scale (GDS))
3. Multidimensional Prognostic Index (MPI)
4. Self-efficacy (Short Scale for Measuring General Self-Efficacy Beliefs (ASKU))
5. Health-related quality of life (European Quality of Life 5 Dimensions 5 Level Version (EQ-5D-5L))
6. Patient-centeredness of care (Patient Assessment of Chronic Illness Care (PACIC))
7. Burden of treatment (Multimorbidity Treatment Burden Questionnaire (MTBQ) and adapted ICAN Discussion Aid)
8. Appropriateness and number of prescribed drugs (criteria of the PRISCUS and FORTA lists)
9. Adherence to prescribed drug therapy
10. Medication literacy
11. Number and days of stays in rehabilitation facilities
12. Number of transfers to “acute” geriatric units
13. Overall survival

On the healthcare system level, the project will elicit barriers, facilitators, and acceptance for the implementation of eliPfad in hospital care and the ambulatory sector.

On the health economic level, the project will investigate the cost-effectiveness of the new model of care from the perspective of the Statutory Health Insurance.

The process evaluation will be guided by the recommendations of the Medical Research Council (MRC) guidance on the process evaluation of complex interventions [38]. It will address the following questions:

1. Is the intervention faithful to the planned model of care?
2. What are the facilitators and barriers to the implementation of the intervention?
3. What is the level of acceptance among patients or relatives, case managers, study nurses, and physicians?

## Trial design {8}

eliPfad is a pragmatic, randomized, controlled, open-label, multicenter trial with one intervention and one control group. The allocation ratio is 1:1. eliPfad is a superiority trial in which the superiority of the new model of care to the current standard of care is to be proven.

## Methods: participants, interventions, and outcomes

### Study setting {9}

eliPfad will be conducted as a multicenter trial in 5 centers with 6 corresponding hospitals and the home environment of each patient. The participating centers/hospitals are the University Hospital Cologne, University Hospital Aachen, Hospital Dortmund, Hospital Herne, and as one center with two corresponding hospitals the St. Franziskus Stiftung in Münster with St. Franziskus Hospital and Hospital Hiltrup. So far, two physician networks in Cologne and Münster participate in eliPfad and represent the ambulatory sector. Patients are enrolled into the new model of care eliPfad during a hospital stay (index hospital stay) which continues after discharge in the home environment. After discharge, patients will be visited at home by the “case manager” (introduced later).

### Eligibility criteria {10}

The inclusion criteria are:

1. Emergency hospital admission or transfer to a normal internal medicine ward in the form of:
  - Direct admission from home / the referring GP
  - Admission from the emergency department
  - Transfer from another hospital (reason for admission: internal medicine)
  - Transfer from another ward within the hospital with initial admission due to an internal disease
2. Age ≥55 years
3. Multimorbidity (presence of at least 3 long-term health conditions)
4. At least one of the following index diagnoses:
  - Heart failure (NYHA I-IV)
  - Chronic kidney disease (CKD)
  - Chronic obstructive pulmonary disease (COPD)
  - Diabetes mellitus (DM)
  - Peripheral artery disease (PAD)
  - Coronary heart disease (CHD)
  - Arterial hypertension (AH)
5. High risk of unplanned readmission (risk assessment by Multidimensional Prognostic Index (MPI) ≥0.34)
6. Written informed consent, documented by signature, after sufficient reflection period. Patient-informed consent cannot be obtained during a medical emergency

The exclusion criteria are:

1. Patients admitted to the hospital due to a non-internal disease (this includes the reason for initial admission to the referring hospital)
2. Patients likely to be released from hospital within 2 working days
3. Residents of nursing facilities, home-based 24-h care, predominantly bedridden patients
4. Lack of rehabilitation potential according to the attending physician’s assessment
5. Insufficient command of German language among patients and relatives/friends
6. A higher degree of visual and/or hearing impairment, if no relatives available
7. Diseases with a life expectancy of less than 6 months
8. Desired rehabilitation on days 1 to 42 after discharge from index hospitalization
9. Travel distance from participating hospital to patient’s home 50 km or more (exceptions based on individual decision are possible)
10.  Patients with full private health insurance
11.  Patients who are in a dependent/employed relationship with the investigators
12.  Lack of capacity to consent
13.  Placement in an institution by court or official order

### Who will take informed consent? {26a}

The physician responsible for the trial in each hospital will inform eligible patients about the trial and obtain informed consent.

### Additional consent provisions for collection and use of participant data and biological specimens {26b}

Informed consent for the collection and evaluation of patient data and biological specimens will be obtained during the main information process. No additional consent provisions are planned.

## Interventions

### Explanation for the choice of comparators{6b}

The control group will be treated according to the current standard of care. eliPfad provides additional care none of which is currently implemented in the German health care system. It is therefore reasonable to compare eliPfad to the current standard of care to justify its implementation in case of proven superiority.

### Intervention description {11a}

#### Course of the intervention

Patients in the intervention group will be treated in the same medical way as the control group. In addition, these patients will receive eliPfad care. The practical implementation is carried out using cross-sectoral care by an interdisciplinary team, coordinated by a specially trained case manager. The case manager functions as a central point of organization between the different caregivers and the patient. They additionally act as a continuous contact for the patients for any kind of eliPfad-related problems or questions. The case manager sets up an electronic medical record for every patient in the intervention group. Every care provider involved in the treatment of the patient during eliPfad (GP, hospital physician, pharmacologist, physiotherapist, etc.) is granted access to this electronic patient file and can contribute by uploading diagnostic and treatment information themselves. This allows care providers to access the most recent health information about the patient at any time. The case manager informs the patient’s GP and—if consented—relatives/friends about the patient’s participation in eliPfad who act as an important source of support after discharge from the hospital.

A personalized treatment plan is developed according to the criteria of minimally disruptive medicine [7, 39]. In its preparation involved are physicians (hospital and GPs) as well as physiotherapists and pharmacists. The treatment plan’s core element is the consideration of the patient’s wishes and individually agreed treatment goals. Another part of this treatment plan is individually selected physiotherapeutic exercises. All treatment plans are uploaded to and accessible for all caregivers in the electronic medical record.

Beneficial to their self-management, patients are supported by a “smart assistant,” consisting of a tablet and electronic smart devices, which enable the patients to measure their vital parameters themselves when at home. Monitored vital parameters include blood pressure, pulse, body temperature, body weight, step count, saturation of peripheral oxygen, and, if necessary, blood sugar. Threshold values for these vital parameters are determined for each patient individually in line with evidence-based medical guidelines. The measured vital parameters are transferred to the electronic medical record once daily. Videos of the exercises taught by the physiotherapists during the hospital stay are retrievable on the tablet and allow patients to practice on their own after discharge. Furthermore, the tablet provides the patients with information about their diseases and the corresponding rationales for the specific treatments, the correct frequency and intake of the prescribed drugs, and general information on the importance of healthy nutrition and physical activity. Moreover, the tablet automatically reminds patients of their medication schedule via notification and requests digital confirmation of the intake. Finally, patients are asked to fill in a digital health diary, assessing the development of their subjective well-being, the occurrence and severity of various symptoms, and the level of self-efficacy.

Before patients are discharged from the hospital, the case manager coordinates the seamless continuation of the treatment plan in the ambulatory setting. In the first week after discharge, the case manager visits the patients in their home environment and ensures that all devices work properly. The patients continue their therapeutic exercises at home, supported by the videos on the tablet, and measure their vital parameters daily. The case manager inspects the vital parameters twice weekly and ascertains that none of the predefined thresholds is exceeded. The patient’s subjective well-being is ensured, additionally to the patient’s reported answers from the health diary, through weekly video calls between the case manager and the patient. The main contact person for health and medical-related questions during the period at home is the patient’s GP. Measured vital parameters along with other relevant health care information of each patient are reported once weekly via standardized reporting sheets in the electronic patient file to their GP.

In case the vital parameters exceed the predefined thresholds, or the patient discloses worsening of their condition, the patient’s GP will be contacted to decide on how to proceed with the patient’s treatment. If deemed necessary, the GP can ask the case manager to organize an additional eliBoard with the consulting physician of the discharge hospital. During this conference, an adaptation of the current treatment plan will be made to prevent an imminent readmission of the patient.

The intervention ends 6 weeks after the initial discharge. The case manager will visit the patient again for the final assessment and will collect the tablet and the medical devices.

#### Time frame of the intervention

Patients are enrolled within two working days after admission to an internal ward (see eligibility criteria above) and the hospital part of eliPfad begins. After discharge from the index hospital stay, patients will be treated in eliPfad for 42 days in the ambulatory setting. This time frame is fixed and will not be prolonged due to planned or unplanned hospital stays within the 42 days. Should the patient be admitted to a hospital within 42 days, the intervention would pause during that period and resume afterward—given that the 42nd day after the initial discharge is not yet reached. A follow-up visit is planned 6 months after the discharge from the index hospital stay.

### Criteria for discontinuing or modifying allocated interventions {11b}

Each patient can decide to stop participation in the trial at any given point in time without any further explanations. A distinction is made here as to whether patients wish to withdraw from the intervention, the assessments or the consent to the transfer of health insurance data. If patients only wish to withdraw from the intervention and the assessments, the health insurance data used to determine the primary outcome can still be collected. The three home visits of the case manager should ideally take place until the end of the following week after discharge from the index hospital stay and subsequently 42 days (+ 5 working days) and 180 days (+ 10 working days) after the initial discharge. In case of unforeseen events (unplanned hospital admissions), the visits will be rescheduled to the next possible date.

### Strategies to improve adherence to interventions {11c}

Adherence to the intervention is crucial and several procedures have been implemented to ensure it. During the time in the hospital, the case manager educates the patient (and/or his caretakers) about his/her chronic conditions and the self-management of their chronic conditions. Additionally, he/she trains the patient in the use of the “smart assistant.” Furthermore, the physiotherapist teaches the patients individual exercises which they can perform independently or with the help of the “smart assistant” at home. This facilitates that the patients (and his/her caretakers) understand the personal benefits of the intervention. The patient is provided with an individualized home exercise plan and it is automatically recorded whether and how long the patient watches the videos. Furthermore, participation in the outpatient physiotherapeutic treatments is documented on the tablet by the physiotherapist. In case of insufficient use of the videos or participation in physiotherapeutic treatments, the case manager can discuss possible problems with the exercises and motivate patients to conduct them. Further measures are not implemented because this trial aims at assessing the effectiveness of eliPfad to provide reliable evidence to decide on whether to include it in standard care for patients. Any additional, trial-specific incentives for patients in the intervention group would flaw the effects and bias the overall results.

### Relevant concomitant care permitted or prohibited during the trial {11d}

The only restriction for patients regarding concomitant care is the use of rehabilitation measures within the intervention period of eliPfad in the ambulatory setting. Patients who clearly state that they want to participate in rehabilitation measures before the 42nd day after discharge are not eligible for eliPfad (see “Eligibility criteria”). The reason is that eliPfad aims at reducing the number of rehabilitation measures because participating in eliPfad is considered to be a rehabilitation measure itself.

### Provisions for post-trial care {30}

Patients in the intervention group will be regularly treated by their GP in the same way as the control group after the final assessment by the case manager. No further interventions after the 6 weeks in the ambulatory setting are planned.

### Outcomes {12}

#### Time points, data collection, and outcomes

Four time points (T) are defined which are accompanied by timely scheduled assessments (A). All assessments are carried out by members of the study team in person to ensure the completeness of the data. Pretests of the single questionnaires and the entire data collection assessment were conducted to test duration and feasibility for the patient population. Depending on the visit, these assessments are carried out either in hospital or at the patient’s home:

1. T0/A0: Baseline, immediately after randomization and up to 2 working days after admission to the internal ward (in the hospital).
2. T1: Discharge day from the index hospital stay. A1 can be performed up to 3 days before scheduled discharge (in the hospital) or up to 3 days after T1 (in the patient’s home).
3. T2: End of the intervention phase, 42 days after T1. A2 can be performed up to 5 working days after T2 (in the patient’s home); if A2 cannot take place in this period (hospital admissions, etc.), A2 can still be collected on day 60 at the most, counted from discharge from the index hospitalization.
4. T3: 180 days after T1. A3 can be performed up to 10 working days after T3 (in the patient’s home).

The primary outcome is the number of unplanned rehospitalizations over 180 days after the end of the index hospital stay. Unplanned inpatient readmission is defined as the admission of a previously discharged patient due to unplanned events or deterioration of health that can no longer be treated in the outpatient setting. The rehospitalization frequencies are determined using collaborating health insurance companies’ data. In addition, at A3, the patients are actively questioned by the case manager about possible hospital stays in the last 180 days.

The secondary outcomes are the variables required for the testing of the secondary hypotheses. The variables collected, when they are collected and the type of source are listed below:

The following data will be obtained by A2 at the latest:

- Duration of index hospital stay
- Duration of stays in intensive or intermediate care units during the index hospital stay
- Duration of stays in other departments within the index hospital stay, if these departments are not part of the local eliPfad team
- Transfers to external hospitals including geriatric facilities
- Length of stays in external hospitals including geriatric facilities after the index hospital stay
- Transfers to rehabilitation facilities
- Duration of stays in rehabilitation facilities

The following data are collected using collaborating statutory health insurance companies’ data at A3:

- Frequency and type of use of services in outpatient and inpatient care, drug prescription, remedies, and aids between T1 and T3
- Number of rehabilitation services between T1 and T3
- Number of transfers to “acute” geriatric units between T1 and T3
- Length of stay in inpatient facilities (incl. hospitals and rehabilitation facilities)
- Death between T1 and T3
- The sum of costs based on the above-defined service areas of collaborating health insurance companies’ data between T1 and T3

The following data will be collected by questioning the participants (if necessary, the relatives, the treating physicians) at all survey points (A0–A3):

- Quality of life using EQ-5D-5L
- Depression using the Geriatric Depression Scale (GDL)
- Geriatric assessment using the Multidimensional Prognostic Index (MPI)
- Timed up and go Test (TUG)
- De Morton Mobility Index (DEMMI)
- Minimal Nutritional Assessment
- Health status using the Self-Rated Health Questionnaire
- Self-efficacy (General Self-Efficacy Short Scale, ASKU)
- Medication plan
- Health literacy
- The personal treatment burden

The following data will be collected at the survey points A0, A2, and A3:

- Patient-centeredness of care using PACIC

The following data will be collected at A1, A2, and A3:

- Questioning about personal therapy goals / self-management

The following data are recorded at A2 and A3:

- Actual number of drugs taken by the patient at home using photo documentation

The following laboratory values are documented as original values at A0 and A1:

- Determination of index diagnosis-specific blood parameters. The standard laboratory panel includes the following: blood count, glucose, sodium, potassium, chloride, phosphate, creatinine, urea, uric acid, ASAT, ALAT, gamma-GT, AP, bilirubin, total protein, albumin, lipase, LDH, CK, NT-proBNP, CRP, HbA1c
- 24-h urine collection: volume, sodium, potassium, chloride, urea, uric acid, creatinine, albumin, total protein, and osmolality to determine dietary salt intake, urea appearance, renal function, concentration performance

The following laboratory values are documented as original values at A2 and A3:

- Determination of index diagnosis-specific blood parameters. The standard laboratory panel includes the following: blood count, sodium, potassium, creatinine, urea, uric acid, albumin, NT-proBNP, CRP, HbA1c
- 24-h urine collection: volume, sodium, potassium, chloride, urea, uric acid, creatinine, albumin, total protein, and osmolality to determine dietary salt intake, urea appearance, renal function, concentration performance

All details and methods of data analysis will be documented in a statistical analysis plan (SAP). The SAP shall be finalized before database closure and will be approved by the two principal investigators and representatives of the evaluating statistics departments.

Data of process evaluation will be collected across the implementation of the intervention:

- Enabling and hindering factors from the perspective of the patients or their relatives, physicians, case managers, and study nurses in the implementation of eliPfad using individual interviews 3 months after the start of the study and continuously until the end of intervention.
- Examination of the acceptance of the intervention by patients or their relatives, physicians, case managers, and study nurses.
- Analysis of the quality of the intervention implementation.
- Survey of implementation hurdles in case of a national rollout through a nationwide online survey of general practitioners and specialists in private practice; this information will be collected using a website developed specifically for this purpose.

#### Explanation

The number of rehospitalizations has been selected as the primary outcome because revolving door effects leading to rehospitalizations are responsible for the majority of costs in the treatment of the target population and a common problem in this patient population.

### Participant timeline {13}

The participant timeline is depicted in Table 1.

**Table 1:** Participant timeline. The time points are explained in the main manuscript (see 12)

	**Study period**				
	Enrolment	Allocation	Intervention		Post-intervention
Time point/assessment		T0/A0	T1/A1	T2/A2	T3/A3
**Study participation**					
Eligibility screen	X				
Informed consent	X				
Allocation		X			
**Clinical data collection**					
Evaluation of adverse events (AE)		X	X	X	X
Duration index hospital stay			X		
Duration stays in different wards			X		
Transfer to external hospitals			X		
Transfer to rehabilitation facilities			X		
MPI (geriatric assessment)	X		X	X	X
Health literacy		X	X	X	X
Quality of life/depression/health status/self-efficacy		X	X	X	X
Medication plan		X	X	X	X
Minimal Nutritional Assessment		X	X	X	X
Mobility tests		X	X	X	X
Patient orientation		X		X	X
Personal goals/self-management/personal therapy burden			X	X	X
Photo documentation drugs				X	X
Number of rehospitalizations				X	X
Health economic evaluation					X
Utilization of rehabilitation services					X
Length of stay in inpatient facilities					X
Death					X
**Laboratory data collection**					
24-h collection urine		X	X	X	X
Clinical chemistry		X	X	X	X

### Sample size {14}

Based on literature data and own calculations using data from the collaborating health insurance companies, a relative reduction of the readmission rate of about 25.9% over 6 months after (initial) discharge is expected (see also Berntsen et al, admission rate of 1.89/year corresponding to 0.95 over 6 months) (19) with the following distribution of absolute numbers (#) of readmissions per patient: #0—35%, #1—40%, #2—20%, #3—5% (Ø 0.95). eliPfad will result in a compression of this distribution as listed below: #0—49.5%, #1—33.1%, #2—14.9%, #3—2.5% (Ø 0.704). The Wilcoxon rank sum test was used to calculate the number of cases; by stratification, a power gain is additionally expected. Accordingly, a total of 474 patients (237 per group) are needed to detect this distribution difference with a power of 90% with a two-sided type I error of 5% [40] (R: A Language and Environment for Statistical Computing, Vienna, Austria, package samplesize, call: n.wilcox.ord (power = 0.9, alpha = 0.05, *t* = 0.5, *p* = c(0.35, 0.40, 0.20, 0.05), *q* = c(0.495, 0.331, 0.149, 0.025))). To compensate for up to 50% loss-to-follow-up in the control group and 30% in the intervention group, 474 patients should be randomized to each of the two groups (total 948 = 474/0.5). In the scenario given above, for the evaluation of the primary outcome measure using Poisson regression, the power of at least 90% was confirmed by simulation.

### Recruitment {15}

Enrolment of patients in eliPfad will occur within two working days after admission to an internal ward if the eligibility criteria are met. To achieve efficient recruitment at each site, investigators at participating hospitals will form collaborations with other internistic departments and the emergency ward within their sites, allowing automated identification of suitable patients under privacy regulations. Close patient support will be provided by a case manager who will coordinate the transition from inpatient to outpatient care, assist the patient in implementing the specific elements of eliPfad, and serve as the primary point of contact for the patient during eliPfad. Case managers have been trained as nurses (3 years in Germany) and have worked in this role in a hospital for several years. In addition, they have advanced training as a case manager with additional training specific to eliPfad (certified by the “Deutsche Gesellschaft für Care und Case Management” (German Association for Care and Case Management, DGCC)). This training program comprises 216 h of courses including 3 days of supervision and 150 h of self-study. The case manager at each site routinely receives daily information on the admission of appropriate patients by implementing adequate arrangements for IT-based identification of patients at each hospital. In addition, direct recruitment occurs through the participating wards in each center, which are specifically contacted and queried by the case manager on all business days. The case manager verifies that patients meet or do not meet the inclusion and exclusion criteria and also assesses the MPI for this purpose. Patients who meet all criteria and wish to participate in the trial will be informed about the study by the investigator, including an information pamphlet. Written informed consent is obtained. Primarily, patients of the collaborating health insurance companies are recruited, but patients of other health insurance companies can also be included in eliPfad under a treatment contract.

## Assignment of interventions: allocation

### Sequence generation {16a}

Sequence generation is performed by the electronic data capture system (EDC, secuTrial®), which also provides the electronic case report form (eCRF). The software’s own “Extended Stratified Block without List” is used here, which represents a stratified block randomization. Factors for stratification are center and sex. Because of the 6 centers, there will therefore be 12 strata.

### Concealment mechanism {16b}

The allocation of the patient to the intervention or control group is done by secuTrial® (see above). Only after the inclusion of the patient and fulfillment of all inclusion and exclusion criteria, the patient can be randomized. Since the randomization is done without a list as mentioned above, the allocation can in no way be known before the randomization. Allocation concealment is therefore guaranteed.

### Implementation {16c}

Potentially eligible patients are screened. This verifies that patients meet the inclusion and none of the exclusion criteria. This is performed by the study physician and study nurse. If all criteria are met, the study physician will inform the patient. Once the patient has given informed consent to participate in the study, their data is entered into the eCRF (hosted by secuTrial®). Subsequently, secuTrial® also carries out the randomization, and the study team is directly informed in which group the patient will participate in eliPfad.

## Assignment of interventions: blinding

### Who will be blinded {17a}

Blinding of the participants is impossible given the kind of intervention of this trial. Assessors will not be blinded either, because patients in the intervention group will be in possession of the tablet and the medical devices. During the hospital stay, these items will be in close proximity of the patient and difficult to hide. At A2 it is the assessor’s task to take these items back to the study center after the assessment. Therefore, they need to know which patient is in which group. However, observer bias is not estimated to be a big problem, because all questionnaires are either filled in by the patient directly or require answers that need no interpretation by the assessor such as number of drugs prescribed or weight loss. Nevertheless, to minimize observer bias, all assessors are specially trained. All evaluators will be blinded.

### Procedure for unblinding if needed {17b}

The design is open label with only outcome assessors being blinded so unblinding will not occur.

## Data collection and management

### Plans for assessment and collection of outcomes {18a}

Data for the primary outcome—the number of rehospitalizations 6 months after the discharge from the index hospital stay—will be cleaned from the data provided by the collaborating statutory health insurance companies. The quality of the data provided can be assumed to be high and the data to be complete. Specification of the dataset to be delivered by the health insurance companies will be determined beforehand. Data will be delivered at two time points by the health insurance companies. Data will be cleaned and quality assured. The secondary outcomes will be collected by the case manager or a study nurse during visits to the patient’s home. Most of the secondary outcomes will be assessed using questionnaires. These questionnaires have been validated and produce reliable results and are therefore scientifically accepted.

### Plans to promote participant retention and complete follow-up {18b}

The collection of the data for the primary outcome will be independent of the patient’s cooperation. The information will be provided by the collaborating health insurance companies. Data collection for the secondary outcomes will always be conducted by members of the project team via a personal visit to the patient’s home. Questionnaires will never be sent to patients via mail.

### Data management {19}

Study data are collected via an online electronic data capture system (secuTrial® database) that can be accessed online in a browser-based manner. All collected data are entered directly into the study’s eCRF by the study teams at the centers. An audit trail records the initial entries and any changes made, the time and date of entry, and the username of the person who authorized the entry or change.

The EDC system is accessed via a standard browser on a device connected to the Internet. Password protection ensures that only authorized persons can access the system to view, add, or edit data according to their permissions. Records and documents related to the conduct of this study, including eCRFs, informed consent forms, laboratory test results, and clinical records will be retained for 10 years.

### Confidentiality {27}

A Trust Center is being set up as part of eliPfad. This is located in the study center of the Medical Clinic II for Internal Medicine of the University Hospital Cologne. The persons working there do not participate in any way in the data collection, patient care, or evaluation of eliPfad. The Trust Center is divided into a confidence center and a data management center. Both units are separated from each other in terms of space and personnel.

The confidence center has the non-encoded names, addresses, and the date of enrollment in the project of the patients. This data is needed so that the confidence center can send invitations to individual interviews to patients in the eliPfad project on behalf of the evaluators at the scheduled times.

Name, date of birth, insurance number, and health insurance are required by the confidence center to obtain the insurance billing data from the collaborating statutory health insurance companies. For this purpose, the collaborating health insurers receive from the confidence center a list of their insured persons (name, date of birth, insured person number) with an assigned pseudonym, so that the health insurers can then transmit pseudonymized data to the data management unit. No further data is transmitted to the health insurance companies. For patients who are insured with other statutory health insurance companies, the confidence center requests the patient receipts from their respective health insurance companies. Patient receipts are proof of all individual services financed by the health insurance company. As soon as the patient receipts are available, all personal information is removed and the receipt is given a pseudonym.

To be able to perform these tasks, the confidence center manages the pseudonym keys, i.e., the assignment of the patients’ plain names with address, insurance number, and date of birth to the patients’ pseudonyms used in the project. The electronic storage of the assignment of the pseudonyms to the non-encoded names takes place exclusively on a password-protected stand-alone computer of the trust center, which is located in an access-restricted room.

The second unit in the Trust Center is the data management unit. The main tasks and responsibilities of the data management unit within the trust center are the management of the pseudonymized study and pseudonymized SHI data, as well as the linking of this data and their forwarding to the evaluators.

### Plans for collection, laboratory evaluation, and storage of biological specimens for genetic or molecular analysis in this trial/future use {33}

Urine and blood samples will be collected at A0–A3. Blood samples will be drawn by the case manager or a study nurse of each center. Measurements will be conducted in the laboratory of the respective center.

## Statistical methods

### Statistical methods for primary and secondary outcomes {20a}

The primary analysis set is derived according to the intention-to-treat-principle (treatment policy strategy): All patients who are included in the study and randomized are analyzed in their allocated group. A secondary analysis set is defined according to the hypothetical strategy (per protocol), comprising all included, eligible, and randomized patients who proceed without interruptions (especially transfers) from stationary to ambulant care. The primary null hypothesis is tested using negative-binomial regression of the number of individual readmissions on the group, if it converges to a solution on the collected data, assuming overdispersed data. In case of equidispersion or non-convergence of the negative-binomial regression, Poisson regression is used. Models will include age, sex, study site, offset (log time under risk), and robust variance-covariance estimation (clustered sandwich estimator) (Stata/SE 17.0, StataCorp LLC, College Station, TX, USA; call: poisson/nbreg outcome group, sex age study.site offset(log.time.under.risk) irr vce(cluster patient.id)). Negative-binomial regression is favored if it converges to a solution on the collected data. Other possible confounder will be specified in the SAP after discussion by clinicians and evaluators and variables will be included in the analysis. Time under risk is defined as the time patients spend at home or in a care facility that does not have medical staff on duty. If a patient dies, they will no longer contribute time under risk from this point onwards. In case of underdispersed data a generalized Poisson model is considered, which is capable of dealing with underdispersion. For the main effect group (relative rate reduction), the *p*-value and the associated two-sided 95% confidence interval are determined. The significance level is 5% two-sided.

The regression approach can take into account the shorter time under risk (than 6 months), especially due to loss-to-follow-up or dropout. The main estimator here is the ratio of event rates (defined as the total number of readmissions/sum of risk times) of intervention to the control group.

Secondary analyses models will also include strata and confounder. The frequencies of repeated events like number of rehabilitation services or transfers to acute geriatric units between T1 and T3 (health insurance companies’ data) and time variables like duration of stay in index hospital, intensive care unit, external hospitals, or rehabilitation facilities will be analyzed analogous to the primary endpoint. Patient questionnaire scales will be evaluated by linear mixed models with repeated measurement over time. Time to death will be analyzed by Cox regression.

In general, quantitative target variables are described by the number of valid cases, mean ± standard deviation, and percentiles (0, 25, 50, 75, 100), and qualitative target variables by absolute and relative (%) frequencies, over time.

All details and methods of statistical analysis will be documented in a statistical analysis plan (SAP). The SAP shall be finalized before database closure and will be approved by the two principal investigators and representatives of the evaluating statistics departments.

### Interim analyses {21b}

The intervention in eliPfad consists of several tried and tested procedures, such as regular measurement of vital signs using certified equipment, as well as home physiotherapy exercises. None of these procedures pose a substantial risk to the patient, so there is no indication to carry out interim analyses that could lead to an early stop of the study due to an obvious harm to the patient.

Furthermore, the study is not an adaptive trial in which the sample size might be adjusted after interim analyses.

For these reasons, no interim analyses are planned.

### Methods for additional analyses (e.g., subgroup analyses) {20b}

#### Analyses of safety-related aspects

Adverse events are assessed by relation, severity, and intensity; treatment comparisons are made in categories (e.g., MedDRA codes).

Changes in laboratory values are shown using shift tables; in addition, individual time courses are described and extreme values are marked in lists.

#### Subgroup analyses

Subgroup analyses are performed by sex, age, index diagnoses as defined in the inclusion criteria section, and study center. There is no alpha adjustment for multiple comparisons.

### Methods in analysis to handle protocol non-adherence and any statistical methods to handle missing data {20c}

The occurrence of missing data is minimized as much as possible through careful study planning and execution. For the primary analysis, all individual times under risk are considered, especially for patients lost to follow-up or dropped out. Moreover, since the primary endpoint data is obtained by health insurance companies, we do not expect a relevant amount of missing data for the number of rehospitalizations. Time-to-event analysis and mixed models deal naturally with missing values. For further analyses, missing data are replaced by multiple imputation if necessary and the results obtained are summarized according to Rubin’s rule [41]. The missing values are first replaced under the missing-at-random (MAR) assumption. In addition, not-missing-at-random (NMAR) scenarios can be realized by post-processing multiple substitutions.

### Plans to give access to the full protocol, participant-level data, and statistical code {31c}

Access to the full protocol, participant data, and statistical code may be granted upon request and approval by the steering committee.

## Oversight and monitoring

### Composition of the coordinating center and trial steering committee {5d}

The University Hospital Cologne represents the coordinating center in this trial. The steering committee comprises representatives from the coordinating center, the figus institute, the Gesundheitsnetz Köln Süd, and the Institute of Health Economics and Clinical Epidemiology (IGKE). The steering committee is responsible for recruitment, intervention development, and evaluation. The steering committee will meet every 2 weeks. Data that will be reviewed are foremost data regarding recruitment/enrollment stratified by the different study centers. The committee ensures that enough p1atients are recruited to reach the planned sample size. Strategies will be discussed on how recruitment can be improved in case numbers are not met.

The participating centers/hospitals are the University Hospital Cologne, University Hospital Aachen, Hospital Dortmund, Hospital Herne, and as one center with two corresponding hospitals the St. Franziskus Stiftung in Münster with St. Franziskus Hospital and Hospital Hiltrup.

Two principal investigators (PIs) represent the coordinating center on the steering committee and are responsible for conducting the study at the University Hospital Cologne. In addition, there is a project coordinator who assists in the implementation of the PIs’ decisions and acts as an interface between all professional groups of the coordinating center. Two study physicians are responsible for the daily implementation of the study at the University Hospital Cologne site. They obtain informed consent from suitable patients, develop the treatment plan together with the case manager, and communicate primarily with the physicians in the outpatient area. Furthermore, a case manager is responsible for the patients at the University Hospital Cologne site. She supports the patients in using the tablet and medical devices, draws up the treatment plan and is the primary contact for patients. She also conducts the weekly video calls with the patient after discharge from the index hospital stay. A study nurse is also employed to carry out the patient assessments, enter the data into the database and check it for completeness. Finally, a study nurse carries out the cross-center monitoring but is not otherwise involved in the implementation of the study. At each of the other centers, 1 study physician, 1 case manager, and 1 study nurse are employed for the tasks described above. The members of each center involved in the day-to-day study activities meet once a day to coordinate the day and assign tasks. Each center has hired its own employees and works independently.

### Composition of the data monitoring committee, its role and reporting structure {21a}

The establishment of an official data monitoring committee (DMC) is not planned. However, to ensure data quality, regular checks will be performed during the study by employees of the coordinating center (risk-based monitoring). The risk-based monitoring will be carried out by the coordinating center. The data entered in the electronic data capture system for randomly selected patients from all centers are examined for completeness (missing data) and implausible values. At least one these monitorings at each study center will be on site. The results of the monitoring are communicated to the centers so that they have the opportunity to supplement missing data or correct implausible values. Regular meetings of the assigned employees of the coordinating center with the documentarists, study nurses, case managers, and physicians of the participating study centers serve to optimize the processes and correct existing deficiencies.

### Adverse event reporting and harms {22}

No adverse events (AEs) in the strict sense are expected to be caused by the intervention. However, due to pre-existing (chronic) diseases, numerous complications are possible. In addition, the participants’ underlying diseases may worsen. These complications are not documented separately.

No novel or unapproved methods will be used as part of the intervention. Conceivably, a safety-related event could occur during independent home exercise; for example, a fall during a mobilization exercise. Events such as these and all other events that are not associated with the natural course of the pre-existing (chronic) diseases are documented as an AE. In case that such an event leads to hospitalization or death of the patient, it will be recorded as an SAE. AEs and SAEs will be recorded in the electronic data capture system (secuTrial® database) by the study team. AEs and SAEs will be identified by the study team during the weekly video calls between the case manager and the patient or during the assessments. SAEs will be immediately reported to the sponsor by the study teams.

Due to the open-label character of the trial, the obligation to document safety-related events begins in the intervention group at the time of enrollment and ends after the 6-week outpatient phase following the index inpatient stay at T2. There is no documentation requirement in the control group.

An overview of safety-relevant events will be provided to the ethics committee upon request after completion of the study.

### Frequency and plans for auditing trial conduct {23}

Authorized representatives of national or local authorities will be permitted to inspect or audit facilities and records relevant to this study. In addition, at the steering committee meetings (see item 5d), the progress of the study and emerging problems are discussed, and strategies for solving these problems are implemented.

### Plans for communicating important protocol amendments to relevant parties (e.g., trial participants, ethical committees) {25}

The sponsor and principal investigator may modify or propose modifications to the study protocol. Substantial changes will be made only after deliberation by the ECs. In emergencies, deviations from the study protocol may be made to protect the rights, safety, and welfare of subjects without prior approval of the Sponsor and the ECs. Such deviations will be documented and communicated to the Sponsor and the ECs as soon as possible. All non-substantial changes will be communicated to the ECs.

### Dissemination plans {31a}

The results of this study are intended for open-access publication in high-impact peer-reviewed journals read by medical professionals, as well as those working in the field of public health. In addition, the results will be presented in the form of oral presentations and posters at major relevant national and international conferences.

## Discussion

This trial is the first within the German healthcare system to investigate the effectiveness of a new model of care for multimorbid, frail elderly who are at high risk of readmission after hospitalization. Given the current inadequate care for this vulnerable group of patients, eliPfad could become a new standard of care if it lowers the treatment burden and proves to be (cost-)effective, by leading to fewer readmissions. This way it benefits both society and the German health care system by improving overall quality of care and potentially saving health expenditures.

There are some limitations associated with this trial that need to be mentioned. First, due to the nature of eliPfad, it is not possible to blind patients and investigators. This may lead to observer bias on the part of the investigators and increase the dropout rate of patients who have been assigned to the control group and are no longer willing to participate. To address these issues, standard operating procedures (SOPs) will be established to minimize observer bias, and evaluators will be blinded to the results. The assumed higher dropout rate in the control group will be compensated for by increasing the sample size accordingly to account for the higher dropout rate without losing power for the main objective.

A second limitation is that patients in both groups, i.e., the intervention and control groups, are treated in the same wards at baseline, and patients in the control group may receive similar treatment as those in the intervention group. To avoid this form of contamination bias, SOPs are established for all participating professional groups. In addition, the case manager is not present on the ward regularly, but only to see the patients in the study.

## Trial status

The study protocol (version 1.1, 10 August 2023) has been approved by the ethics committee of the University Hospital Cologne. The trial was registered in the DRKS (German Clinical Trials Registry) on 08/14/2023 under the ID DRKS00031500 (https://drks.de/search/en/trial/DRKS00031500). The study will be conducted following the principles of the Declaration of Helsinki. The first patient was enrolled on 09/01/2023. Recruitment will take place over 2 years and runs until 08/31/2025, accordingly. The end of the study is defined as the date on which the (last) A3 assessment was performed on the last patient, which is expected to be in March/April 2026.

## Data Availability

The participating statutory health insurance companies will provide well-defined data to answer the objectives. Every study center can access and enter the data for the trial participants at their center. The evaluators receive all data in pseudonymized form for the final evaluation. The exact procedure for data collection and data entry, as well as access to the data, will be defined in an agreement between all institutions involved in eliPfad.

## Abbreviations

A: Assessment
ASKU: Short Scale for Measuring General Self-Efficacy Beliefs
CCM: Chronic Care Model
DRKS: Deutsches Register Klinischer Studien
CHD: Coronary heart disease
CKD: Chronic kidney disease
COPD: Chronic obstructive pulmonary disease
DEMMI: De Morton Mobility Index
DGCC: Deutsche Gesellschaft für Care und Case Management
DM: Diabetes mellitus
DMC: Data monitoring committee
eCRF: Electronic case report form
EDC: Electronic data capture
e-ePA: Cross-sector electronic patient record
EQ-5D-3L: European Quality of Life 5 Dimensions 3 Level Version
FORTA: Fit fOR The Aged
G-BA: Gemeinsamer Bundesausschuss
GDS: Geriatric Depression Scale
GP: General practitioner
MAR: Missing-at-random
MDM: Minimal disruptive medicine
MPI: Multidimensional Prognostic Index
MRC: Medical Research Council
NMAR: Not-missing-at-random
NYHA: New York Heart Association
PACIC: Patient Assessment of Chronic Illness Care
PAD: Peripheral artery disease
PI: Principal investigator
RCT: Randomized controlled trial
SAP: Statistical analysis plan
SOP: Standard operating procedure
T: Time point
TUG: Timed up and go Test
